# Characterization of the complete chloroplast genome sequence of wetland macrophyte *Typha orientalis* (Typhaceae)

**DOI:** 10.1080/23802359.2019.1698348

**Published:** 2019-12-12

**Authors:** Zhen-Dian Liu, Xiong-Li Zhou, Hai-Ying Ma, Yu-Qiang Tian, Shi-Kang Shen

**Affiliations:** a School of Life Sciences, Yunnan University, Kunming, Yunnan, China;; b School of Ecology and Environmental Sciences & Yunnan Key Laboratory for Plateau Mountain Ecology and Restoration of Degraded Environments, Yunnan University, Kunming, Yunnan, China;; c Faculty of Geographical Science, Center for Human-Environment System Sustainability (CHESS), State Key Laboratory of Earth Surface Processes and Resource Ecology & School of Natural Resources, Beijing Normal University, Beijing, China

**Keywords:** *Typha orientalis*, aquatic plant, lakeside, phylogenetic, chloroplast genome

## Abstract

*Typha orientalis* is an important wetland macrophyte native to the eastern parts of Asia and Oceania. Herein, the complete chloroplast genome of this species was assembled and characterized using whole-genome next-generation sequencing. The complete chloroplast genome showed a circular genome of 160,969 bp size with 36.6% GC content. The genome is of typical structure and contains a pair of inverted repeat (IR) regions with 26,691 bp, separated by one large single-copy (LSC) with 89,118 bp, and one small single-copy (SSC) regions with 18,469 bp. The genome contained 132 genes, including 86 protein-coding genes, 38 tRNA genes, and 8 rRNA genes. A phylogenetic tree reconstructed based on 15 chloroplast genomes reveals that *T. orientalis* is most related to *Typha latifolia.*

Cattails (*Typha* spp., Typhaceae) are globally ubiquitous wetland macrophytes. They play an important role in nutrients (N, P) removal in wetland habitats. *Typha* consists of approximately 16 species distributed in the world. However, *Typha orientalis* is sparsely distributed in the eastern parts of Asia and Oceania (Wu and Raven 2013). The species exhibits well adaptation in the lakesides and constructed wetlands. Previous studies on *T. orientalis* mainly focused on its ecology (Inoue and Tsuchiya 2009), and genetic information is still limited. Good knowledge of comprehensive genomics information would contribute to the species’ utilization. The present study is the first time to assemble and characterize the complete chloroplast genome for *T. orientalis* using next-generation sequencing technology. Such information will pave the way for future studies on phylogenetic evaluation and utilization of *T. orientalis*.

Fresh leaves of *T. orientalis* were collected from the lakeside of Dianchi in Yunnan Province, China (E102°44′7.57′′, N24°55′44.54′′). The specimen is stored at Yunnan University Herbarium (HYN-SSK190012). Total genomic DNA was extracted using a modified cetyltrimethylammonium bromide (CTAB) method (Doyle 1987). The sequencing library was constructed and quantified, and then the paired-end (PE) libraries were generated using Illumina HiSeq 2500 platform. The whole-genome sequencing was conducted by Softgene (Beijing, China). We assembled the short reads into contigs using SPAdes, connected all contigs with Bandage, and manually removed redundant contigs. We mapped reads to the genome to check, proofread, and patch and finally obtained cycle complete plastomes. The chloroplast (cp) genome was annotated through DOGMA (Wyman et al. 2004), and the boundaries of start and stop codons, and intron/exon were checked manually using Geneious version 8.1.4. We confirmed all tRNA genes using online tRNAscan-SE (Schattner et al. 2005). The final complete plastomes were deposited in GenBank with an accession number MN602748.

The cp genome of *T. orientalis* is a circular molecule of 160,969 base pairs (bp), with a pair of Inverted Repeats (IR) of 26,691 bp, separated by a large (LSC, 89,118 bp) and a small single copy (SSC, 18,469 bp) regions. The overall GC content of *T. orientalis* cp genome is 36.6% and the corresponding values in LSC, SSC, and IR regions are 34.4, 30.6, and 42.4%, respectively. The cp genomes were annotated with 132 genes, including 86 protein-coding genes, 38 tRNA genes, and 8 rRNA genes. A total of 77 simple sequence repeats (SSRs) were detected using the online software MISA (http://pgrc.ipk-gatersleben.de/misa/. Beier et al., 2017). The numbers of mono-, di-, tri-, tetra-, penta-, and hexanucleotide SSRs are 32, 23, 3, 16, 1, and 2, respectively.

To reveal the systematic position of *T. orientalis*, we performed a phylogenomic analysis using the chloroplast genomes sequences of 15 species (*Carex neurocarpa* as outgroup) in PAUP version 4.0a with 1000 bootstrap replicates (Swofford 2002). The phylogenetic tree indicated that *T. orientalis* has closer relationship with *Typha latifolia* than other species with a 100% bootstrap value (Figure 1). This study will provide valuable genomic resources for revealing the species’ phylogeny, exploring genetic variations, and designing utilization strategy.

**Figure 1.**
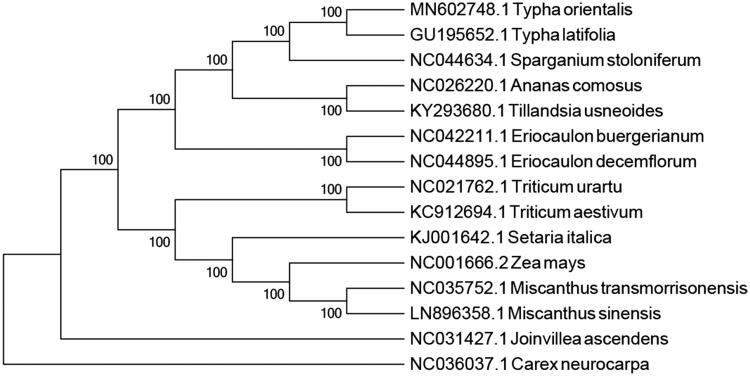
Phylogenetic position of *Typha orientalis* based on the complete chloroplast genome sequences of 15 species. Bootstraps are shown next to the node.
